# Supplementary material for: Mucosal IFNλ1 mRNA-based immunomodulation effectively reduces SARS-CoV-2 induced mortality in mice
Source: EMBO Rep. 2024 Jul 26;25(9):7. doi: 10.1038/s44319-024-00216-4 (PMC11387833; doi:10.1038/s44319-024-00216-4)
Supplement: Supplementary file 7 — Expanded View Figures [file 44319_2024_216_MOESM7_ESM.pdf]

Expanded View Figure

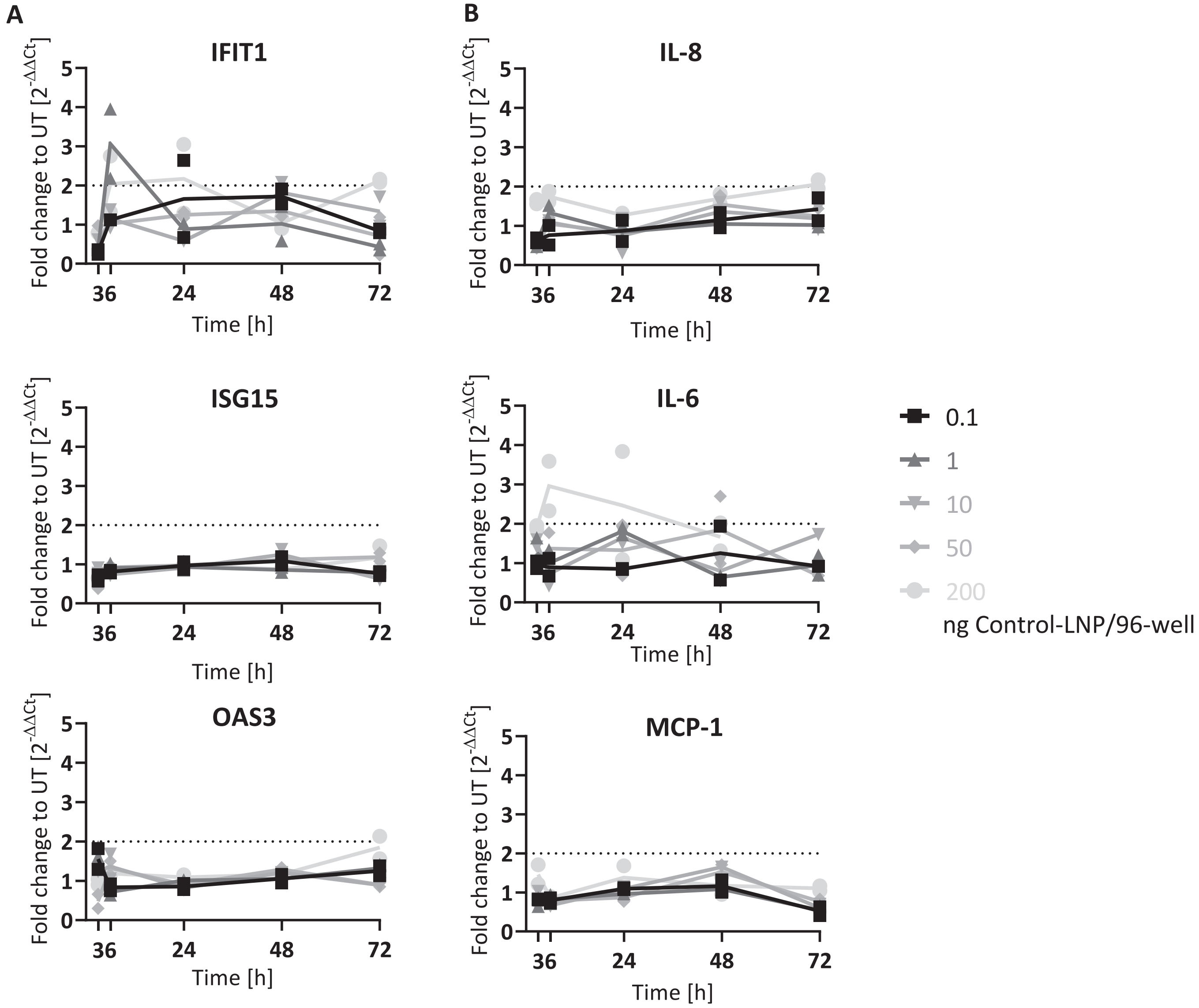

Figure EV1. Single-dose treatment of A549 cells with Control-LNP leads to no or very low target gene induction and no cytokine induction.

A549 cells were treated with Control-LNP ($n = 2$ biological replicates). At 6 and 24 h post treatment medium was exchanged (**A**) Target gene and (**B**) cytokine expression in A549 cells. Data information: data points show single values and mean. Dotted line at $y = 2$: fold changes below 2 are not considered as gene induction. Missing data points reflect Cq values above the set cycle threshold. Source data are available online for this figure.

 © The Author(s)
